# Modulation and Predictors of Periprosthetic Bone Mineral Density following Total Knee Arthroplasty

**DOI:** 10.1155/2015/418168

**Published:** 2015-02-08

**Authors:** Anett Mau-Moeller, Martin Behrens, Sabine Felser, Sven Bruhn, Wolfram Mittelmeier, Rainer Bader, Ralf Skripitz

**Affiliations:** ^1^Department of Orthopaedics, University Medicine Rostock, Doberaner Straße 142, 18057 Rostock, Germany; ^2^Department of Exercise Science, University of Rostock, Ulmenstraße 69, 18057 Rostock, Germany

## Abstract

Total knee arthroplasty (TKA) leads to a loss of periprosthetic bone mineral density (BMD). Great importance is attached to the prevention of periprosthetic bone loss with a view to ensuring a long service life of the prosthesis. In order to provide appropriate recommendations for preventive movement therapy measures to combat peri-implant bone loss, it is necessary to know the predictors of periprosthetic BMD. The aim of this study was (1) to determine the change of periprosthetic BMD of the femur and tibia and (2) to analyse the effects of different predictors on periprosthetic BMD. Twenty-three patients with primary TKA were evaluated 10 days and 3 months postoperatively. The data analysis comprised (1) the change in periprosthetic BMD from pretest to posttest and (2) the correlations between BMD and the variables isometric maximum voluntary force, lean mass, physical activity (step count), and BMI using multiple linear regression and structural equation modelling (SEM). BMD of the distal femur was significantly reduced by 19.7% (*P* = 0.008) 3 months after surgery, while no changes were found in BMD of the tibia. The results of SEM demonstrate that 55% of the BMD variance was explained by the model (χ^2^ = 0.002; *df* = 1; *P* = 0.96; χ^2^/*df* = 0.002; RMSEA < 0.01; TLI = 1.5; CFI = 1.0). A significant direct effect was only evidenced by the variable lean mass (*β* = 0.38; *b* = 0.15; SE = 0.07; C.R. = 2.0; *P* = 0.046). It can be assumed that a large muscle mass with accompanying distribution of high mechanical load in the bones can contribute to local changes of periprosthetic BMD. Concrete recommendations for preventing peri-implant bone loss therefore include exercises which have the aim of maintaining or building up muscle mass.

## 1. Introduction

Total knee arthroplasty (TKA) leads to a decrease in periprosthetic bone mineral density (BMD). The peri-implant bone loss is attributed, amongst others, to stress shielding, osteolysis as a result of abrasion, loosening of the implant, and osteonecrosis [1]. In the region of the distal femur the greatest decline in periprosthetic BMD has been observed within the first 3 months after surgery [2, 3]. The phase of rapid resorption is followed by a slower bone loss, which has been shown to last until at least the 7th year after surgery [2–7].

The load distribution in the bone is modulated following TKA, which results in stress-adaptive bone remodelling (stress shielding effect) [8–10]. This stress shielding effect is viewed as the primary determinant of BMD decline in the early postoperative phase. A bone atrophy of up to 36% has been reported for the anterior region of the distal femur [1–8, 11–21]. Results relating to the periprosthetic BMD in the region of the proximal tibia contradict each other. Some studies documented no change [6, 17, 22], others a decrease [4, 5, 19, 22–24], and others still an increase in BMD [23, 25].

Van Loon et al. stated in their review article that periprosthetic bone loss caused by stress shielding does not, in general, induce any symptoms and clinical problems [1]. However, in various studies it was hypothesised that this effect can lead to a loosening of the implant and can encourage periprosthetic fractures [12, 20, 22, 26]. To our knowledge, there is no evidence for this correlation, even though the empirically demonstrated negative relationship between BMD and fracture risk supports this theory [27, 28]. The occurrence of periprosthetic supracondylar fractures of the femur following TKA is low, with an incidence of 0.3% to 2.5% [29]. Nevertheless, great importance is attached to preventing periprosthetic bone loss with a view to ensuring a long service life of the knee endoprosthesis.

The periprosthetic bone loss can be reduced with the use of bisphosphonates [30]. However, apart from the proven positive effects of medicinal treatment on periprosthetic BMD, the effect of movement therapy measures remains unclear. The Cochrane Review of Howe et al. established that high-intensity exercise contributes to an increase in the BMD of the femoral neck of postmenopausal women with osteoporosis [31]. But the effect of different exercise interventions on periprosthetic BMD has not yet been studied. In order to be able to recommend appropriate movement therapy measures to prevent peri-implant bone loss, it is necessary to determine the most important predictors of periprosthetic BMD. In this regard, the variables of particular interest are those whose severity can be modified by different exercise interventions. There are a large number of scientific studies which describe the effect of different predictor variables on BMD and the results vary, depending on the anatomical regions studied [32–42]. However, no studies exist to date on the predictors of periprosthetic BMD following TKA.

Consequently, the present study analysed two aspects of periprosthetic BMD following TKA: (1) modulation of periprosthetic BMD and (2) predictors of periprosthetic BMD.

As already mentioned, modulation of periprosthetic BMD following TKA has been extensively investigated. Based on the body of published literature, a decrease in BMD—in particular in the region of the distal femur—was expected [1–8, 13–23]. The results of the BMD of the proximal tibia are contractionary and the present study may thus provide further evidence for a modulation in this region. In addition, the study analysed, for the first time, the effect of different predictors on periprosthetic BMD. In this regard, variables were selected whose severity can be modified by exercise interventions and for which a relevant contribution to variance clarification of the BMD had been established for other anatomical regions [32–39, 42–47]. Thus, the effects of isometric maximum voluntary force of the quadriceps muscle (iMVF), lean mass of the thigh, physical activity (step count), and body mass index (BMI) on BMD were analysed. Based on recent literature findings, we hypothesised periprosthetic BMD to be positively correlated with lean mass [32–38], iMVF [32, 39, 42], step count [43], and BMI [41, 42, 44].

## 2. Materials and Methods

The prospective longitudinal study was carried out in the Department of Orthopaedics, University Medicine Rostock, Germany, from August 2011 to April 2012. The study was approved by the Ethical Review Committee of the University of Rostock (A 2009 25).

The participants suitable for inclusion in the study were osteoarthritic patients who had undergone TKA, were aged between 50 and 80, and had a BMI of less than 40. Patients with a total knee endoprosthesis on the contralateral side or a total hip endoprosthesis were included in the study if the operation had taken place more than 1 year before. The following exclusion criteria were defined: musculoskeletal and neurological disorders, metabolic bone disease, an operation planned within the next 12 months, and pain or functional restrictions which would prevent patients from taking part in physical examinations. Prior to taking part in the study all patients signed a declaration of consent. A period of 9 months was allowed for recruiting patients.

**Figure 1:**
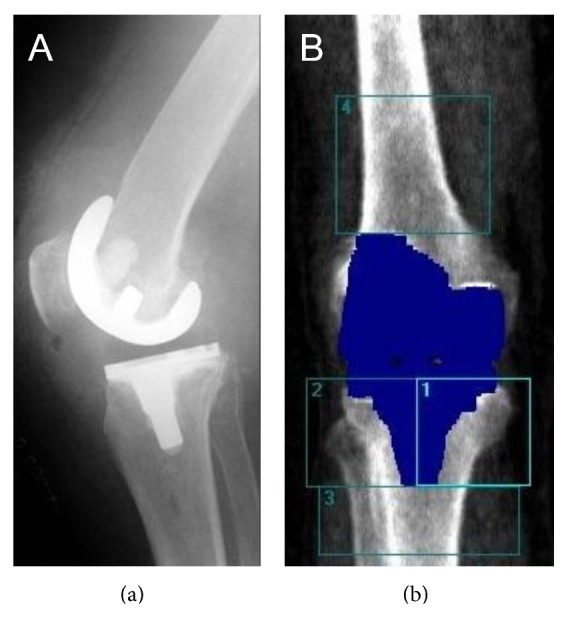
(a) Lateral X-ray scan of the implant and (b) anterior-posterior dual-energy X-ray absorptiometry scan with the periprosthetic regions of interest (ROI) for analysing bone mineral density of the tibia (1 = ROI-T1; 2 = ROI-T2; 3 = ROI-T3) and femur (4 = ROI-F4).

All patients had the same implant (Multigen Plus, Lima-Lto, San Daniele, Italy (Figure 1(a))) fitted using the standard surgical approach for TKA (medial parapatellar approach) [48]. Three different surgeons performed the surgery. Postoperatively patients received continuous peridural analgesia or femoral nerve block. Additionally, a 3-step analgesia was performed to provide optimal pain relief with (1) indomethacin (25 mg), (2) metamizol, and (3) paracetamol. All patients underwent full-weight bearing with two crutches beginning on the second postoperative day.

The patients were examined over a period of 3 months. The study design encompassed 2 measurement points in time: pretest (10 days after surgery) and posttest (3 months after surgery). The data analysis comprised, in the first step, the change in periprosthetic BMD of the femur and tibia from pretest to posttest. In a second step, the correlations between BMD and the variables iMVF, lean mass, step count, and BMI were analysed. Modulation and predictors of periprosthetic BMD were evaluated 3 months after surgery, as the greatest peri-implant bone loss has been observed within this period [2, 3]. The data were collected by the same investigator.

### 2.1. Periprosthetic Bone Mineral Density

The BMD was measured using dual-energy X-ray absorptiometry (DXA) (Lunar Prodigy densitometer, General Electric (GE) Medical System Lunar, Madison, WI, USA). The patient was placed in the supine position on the scanning bed and the operated leg was held in place by an adjusting device. The anterior-posterior scan commenced 15 cm proximal to the superior edge of the patella. The scan lasted 30 s in “small animal/research mode” (76 kV; 0.15 mA; 1.8 *μ*Gy; field of view: 25 cm (*L*) × 20 cm (*W*)). Prior to each test, the quality assurance procedure was carried out using a cuboid-shaped calibration body (200 × 130 × 60 mm). With the help of the Lunar enCORE software (2007 Version 11.40.004) the BMD for four manually defined anatomical regions of interest (ROI) of the operated leg was calculated following the approach of Abu-Rajab et al. and Gazdzik et al.: tibia (ROI-T1, ROI-T2, ROI-T3) and femur (ROI-F4) (Figure 1(b)) [11, 49]. The influence of the metal implant on measurement was eliminated with regard to the software by using an algorithm.

*Precision BMD.* Within the context of longitudinal studies of serial BMD measurements, the International Society for Clinical Densitometry recommends the calculation of the precision error (root mean square standard deviation, RMS SD in g/m^2^, or coefficient of variation, CV%) and the least significant change (LSC = CV% × 2.77) [50]. This was not possible as part of this study, as the additional radiation exposure of patients could not be ethically justified. However, the information on the precision error with DXA measurements does vary considerably. The BMD measurement of the femur with the Lunar Prodigy densitometer demonstrated a high degree of precision, with a CV% of 0.9% [51]. Nevertheless, we did not measure LSC; thus the selection of ROIs and also the determination of LSC followed the approach of Gazdzik et al. [49]: ROI-F4: 0.10 g/cm^2^ (13.63%), ROI-T1: 0.14 g/cm^2^ (14.62%), ROI-T2: 0.13 g/cm^2^ (15.04%), ROI-T3: 0.16 g/cm^2^ (14.15%).

### 2.2. Isometric Maximum Voluntary Force

The measurement of iMVF was carried out on a custom-made force measurement system. It is designed according to the principle of a seated leg press and allows the seating position to be individually adjusted. The measurement of the iMVF of the quadriceps muscle was performed unilaterally with hip joint (90°), ankle joint (90°), and knee joint (60–70°; 0° = full extension) all at constant angles. The patients were instructed to fold their arms across their chests and to press with the whole foot isometrically as forcefully as possible against a panel for 3 s. The patients were given verbal encouragement throughout the execution of the movement. An observer checked that the movement was executed without any visible countermovement. The patients had three attempts for the purposes of familiarisation, followed by three attempts under test conditions. The pause time between the tests was 1 min. The signal was captured with a KM40 force sensor (ME-Messsysteme GmbH, Hennigsdorf, Germany) and preamplified with the GSV3 (ME-Messsysteme GmbH, Hennigsdorf, Germany). The signals were filtered (Lancosh FIR; Lowpass: 50 Hz) with the aid of the Telemyo 2400T G2 8-channel EMG telemetry system manufactured by Noraxon Inc. (Scottsdale, Arizona, USA) and digitalised at a frequency of 3 kHz. The data were analysed using MyoResearch Master Edition 1.06 XP software (Noraxon Inc., Scottsdale, Arizona, USA). The mean value of the three test results was used for the data analysis.

Before the leg press dynamometer was used for patient evaluation, the intrasession reliability for the iMVF was assessed. All statistical procedures were performed using SPSS (Version 20.0, SPSS Inc., Chicago, IL, USA) and a spreadsheet for calculating reliability [52]. The results are presented in Table 1. The iMVF demonstrated high intrasession reliability. The CV% for iMVF was small but slightly above 5%, indicating moderate reliability. The percentage change in the mean between the trials was −2.3% (95% CI: −6.0 to 1.4%). No significant intrasession difference was observed.

### 2.3. Lean Mass

The lean mass was measured using DXA (Lunar Prodigy densitometer), with low exposure to radiation and a minimal radiation dose (<10 mSv; 76 kV; 0.15 mA; 0.4 *μ*Gy) [53]. The patient was placed in a supine position on the scanning bed. The whole-body scan took 6-7 min in standard mode. Prior to each test, the quality assurance procedure was carried out using a cuboid-shaped calibration body (20 × 130 × 60 mm). Using the Lunar enCORE software, the body composition was calculated for one manually determined ROI on the operated leg. The upper limiting line of the ROI runs at an oblique angle above the trochanter major and minor and the lower limiting line horizontally through the knee joint line. The influence of the metal implant on measurement was eliminated with regard to the software by using an algorithm. The relevant parameter is the lean mass of the thigh which was calculated by the Lunar* enCORE* 2007 software. The lean mass represents the bone-free lean mass (appendicular lean soft tissue), composed primarily of the muscle mass as well as other parts such as ligaments, tendons, joint capsules, and meniscal tissue [51]. Levine et al. demonstrated a strong correlation between lean mass determined by DXA and the muscle mass determined by means of computed tomography (*r* = 0.86, *P* < 0.001). Thus, lean mass may be regarded as a valid measure of muscle mass. By comparison with the 4-component model, a systematic error of −0.62% to −4.68% was evidenced when measuring lean mass with the Lunar Prodigy densitometer [54].

### 2.4. Physical Activity

Patients' physical activity was measured over a period of 7 days using the activPAL (PAL Technologies Ltd., Glasgow, UK) activity recording system [55]. This was done by fixing a wire-free sensor on the ventral side of the right thigh in the middle between the knee and the hip with sticking plaster. The patients were instructed to wear the device at all times, with the exception of water-based activities. The activPAL monitor captures the inclination of the femur by means of accelerometry. This meant that every change in position of the patient was documented and the activity pattern of the patients was analysed using the step count. The data were recorded at a frequency of 10 Hz and analysed with activPAL software (Version 7.1.18.).

## 3. Statistical Analysis

### 3.1. Analysis I: Change in BMD

The results from Soininvaara et al. were used for calculating the sample size [6]. The authors provided evidence of a significant decrease in BMD of the distal femur of 13.4% 6 months after surgery (ROI total femoral; pretest: 1.42 ± 0.22 g/cm^2^; posttest: 1.23 g/cm^2^± 0.24 g/cm^2^). The decrease in BMD with a large effect size (*d*_*z*_ = 0.82) lies within the LSC determined for this study. In order to detect a large effect at a significance level of 5% with a probability of 90%, a total of 19 study participants were necessary. Taking into account a drop-out rate of 10%, a resulting sample size of at least 22 patients was needed for this study.

Univariate outliers (boxplots) were eliminated from the data matrix and missing values were filled in using multiple imputation (5 imputed data sets) using the Marco Chain Monte Carlo (MCMC) method [56]. Normal distribution was checked using the Kolmogorov-Smirnov test. The changes in BMD were tested for significance using LSC and paired Student's *t*-test. The change in ROI-T1, ROI-T2, and ROI-T3 was tested using a two-sided *t*-test and the change in ROI-F4 was analysed using a one-sided *t*-test. The significance level was set at *P* ≤ 0.050. Sample size and effect size were calculated with G^*^Power (version 3.1.4.) [57]. The statistical data analysis was carried out with SPSS (version 20.0, SPSS Inc., Chicago, IL, USA). According to Cohen the effect size *d*_*z*_ was interpreted as follows: *d*_*z*_ = 0.20 small effect, *d*_*z*_ = 0.50 moderate effect, and *d*_*z*_ = 0.80 large effect [58].

### 3.2. Analysis II: Predictors of BMD—Multiple Linear Regression

Prior to the analysis of the structural equation modelling (SEM), the classical stepwise linear regression model was calculated. The identification of univariate and multivariate outliers was carried out by means of boxplots and the Mahalanobis distance. Outliers were eliminated from the data matrix and missing values filled in using the full information maximum likelihood estimation (FIML) with AMOS software (version 20.0, SPSS Inc., Chicago, IL, USA) [59]. Linearity and variance homogeneity were checked with a scatter plot between the standardised predicted criterion and standardised residuals. The normal distribution assumption was checked using the Kolmogorov-Smirnov test. The multivariate kurtosis coefficient (Mardia coefficient) and the critical ratio (C.R. value) were used to check the multivariate normal distribution. A multivariate normal distribution may not be assumed if the Mardia coefficient varies significantly from zero. A C.R. value of >1.96 and >2.57 indicates moderate and extreme infractions, respectively, of the multinormal distribution assumption. The multicollinearity was checked using the variance inflation factor (VIF) indicator. A VIF value ≥ 10 was assumed for the cut-off criterion [60]. The coefficient of determination (*R* ^2^) and the standardised (*β*) and nonstandardised (*b*) regression coefficients of the predictors together with the Pearson correlation coefficient (*r*) and squared semipartial correlation (*sr* ^2^) are reported. The statistical data analysis was carried out with SPSS and the effect size calculated with G^*^Power. According to Cohen, the effect size *f* ^2^ was interpreted as follows: *f* ^2^ = 0.02 small effect, *f* ^2^ = 0.15 moderate effect, and *f* ^2^ = 0.35 large effect [58].

### 3.3. Analysis III: Predictors of BMD—Structural Equation Model (SEM)

In addition to the regression analysis, a quantitative assessment of the correlation of the variables was also made using structural equation modelling. Structural equation modelling is a combination of factor, path, and regression analyses and compares the covariance matrix of the hypothesised model with the data observed. It is superior to the classical multivariate methods with regard to both the application possibilities and the quality of the results. Compared to the regression model, with SEM complex relational structures with many individual hypotheses can be checked simultaneously [59].

**Figure 2:**
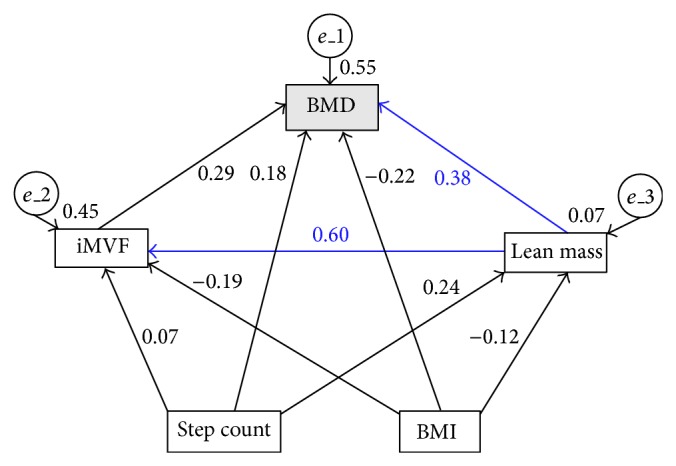
A priori structural equation model. BMD, periprosthetic bone mineral density; iMVF, isometric maximum voluntary force. Chi-square (*χ* ^2^) = 0.002; degrees of freedom (*df*) = 1; *χ* ^2^/*df* = 0.002; root mean square error of approximation <0.01; Tucker-Lewis index = 1.46; comparative fit index = 1.00. Significant path coefficients are indicated in blue (*P* ≤ 0.050).

For the SEM, the direct and indirect effects of the independent variables step count and BMI—as well as the intervening variables iMVF and lean mass—on periprosthetic BMD of the femur were analysed. The SEM with the relationships between the variables was made clear a priori in graphical terms by a path diagram (Figure 2).

The tests for checking the application prerequisites of SEM (linearity, variance homogeneity, multicollinearity, and univariate and multivariate normal distribution) have already been described in Section 3.2. The model fit was assessed on the one hand with the chi-squared test (*χ* ^2^ test) and the quotient from the *χ* ^2^ value and degrees of freedom (*χ* ^2^/*df*). The model fit was also, in line with the recommendations of Hu and Bentler, evaluated using the fit indices root mean square error of approximation (RMSEA), the Tucker-Lewis index (TLI), and the comparative fit index (CFI) [61]. There is a good model fit if the null hypothesis in the *χ* ^2^ test is maintained (*P* ≥ 0.050) [59], *χ* ^2^/*df* ≤ 2 [62], RMSEA ≤ 0.06, TLI ≥ 0.95, and CFI ≥ 0.95 [61]. The statistical data analysis was carried out with AMOS.

## 4. Results

A total of 25 out of 80 patients with primary TKA were enrolled in the study within the 9-month recruitment period. The recruitment of patients was stopped when the scheduled date of closure was reached. Fifty-five of the 80 patients had to be excluded for the following reasons: inclusion criteria not being met *n* = 37, refusal to participate *n* = 9, and being not available for the posttest for reasons of logistics *n* = 9. Two patients could not take part in the posttest for health reasons (drop-out rate: 8.0%). Consequently, 23 patients were included in the data analysis. The demographic and clinical characteristics of the patients are provided (Table 2). None of the patients took any type of antiresorptive medication (e.g., bisphosphonates, hormone replacement therapy, and selective estrogen receptor modulators).

### 4.1. Analysis I: Change in BMD

The target sample size of at least 22 patients was achieved. The data set was incomplete, with a small proportion of univariate outliers (1.6%) and missing values (2.2%) [59]. No significant changes in BMD were found for all 3 ROIs of the tibia (ROI-T1, ROI-T2, and ROI-T3). Both the *P* values (*P* > 0.050) and the interpretation of the results taking the LSC provide no evidence of statistical significance (Table 4). By contrast, the BMD of the distal femur (ROI-F4) was significantly reduced by 19.7% (Table 4). Based on these results, BMD of the distal femur (ROI-F4) was used for further analyses (Analysis II and Analysis III).

### 4.2. Analysis II: Predictors of BMD—Multiple Linear Regression

The data set was incomplete, with a small proportion of univariate outliers (0.9%). Descriptive statistics of the criterion and predictor variables are provided (Table 3). The assumptions on the linearity of the correlations and variance homogeneity of the residuals were fulfilled. With VIF values of <5 (VIF_iMVF_ = 1.80; VIF_step  count_ = 1.07; VIF_lean  mass_ = 1.72; VIF_BMI_ = 1.08), the collinearity statistics did not indicate the existence of multicollinearity. There was no infraction of the univariate normal distribution assumption. The Mardia coefficient of the multivariate kurtosis and the C.R. value, with values of 0.77 and 0.22, respectively, indicate multinormal distribution.

The correlations with the criterion BMD suggested a high declared variance with differing levels of relevance for the predictors (Table 5). Significant correlations with BMD were shown by the variables lean mass (*P* = 0.001) and iMVF (*P* = 0.002). Furthermore, a significant correlation was found between the predictors iMVF and lean mass (*P* = 0.001).

The model for stepwise regression contained, with lean mass, 1 of the 4 predictors and was achieved in 1 step. The predictors iMVF, step count, and BMI were excluded from the model. The model was significant: *F*(1, 21) = 13.652 (*P* = 0.001) and the model equation correlated to *R* = 0.628 with the criterion variable (*R* ^2^ = 0.394; *R* ^2^ adjusted = 0.365; *f* ^2^ = 0.65; power = 0.78). The variance in periprosthetic BMD was predicted to a significant extent only by the variable lean mass (*β* = 0.628; *b* = 0.247; SE = 0.067; *P* = 0.001; *r* = 0.628; *sr* ^2^ = 0.394). Lean mass accounted for 39% of the variance in BMD.

### 4.3. Analysis III: Predictors of BMD—Structural Equation Modelling

For the prediction of the BMD of the femur, the independent variables step count and BMI as well as the intervening variables iMVF and lean mass were taken into account. The prerequisites (linearity, variance homogeneity, multicollinearity, and univariate and multivariate normal distribution) for the use of SEM had been met and have already been described in Section 4.2. The estimation of the parameters for the SEM was made using the maximum likelihood method [59]. Figure 2 shows the a priori SEM for predicting the periprosthetic BMD of the femur. The arrows make clear the causal relationships and direction of effect between the variables. The direction and strength of the direct effects are described through the standardised partial regression coefficients *β*. The value for the *R* ^2^ is shown above each variable and it indicates the proportion of the variance explained by regression. The residual path e leads to every dependent variable. This shows the influence of the estimation errors and/or external variables to the model. The total standardised and nonstandardised regression coefficients of the predictors together with the results of the significance tests are provided (Table 6) and represent the sum of direct and indirect effects. The standardised indirect effects (Table 7) make clear the effect of one variable on another as mediated by an intervening variable.

The results of the *χ* ^2^ test and the fit indices indicate that the a priori model appears to be a good fit to the data: *χ* ^2^ = 0.002, *df* = 1, *P* = 0.960, *χ* ^2^/*df* = 0.002; RMSEA < 0.01; TLI = 1.46; CFI = 1.00. The results demonstrate that 55% of the variance in BMD is explained by the variables iMVF, lean mass, step count, and BMI. A significant direct effect was only evidenced by the variable lean mass which accounted for 38% of the variance in BMD. In addition, the results show that the intervening variable iMVF is significantly determined by lean mass (Table 6; Figure 2). No modifications were made to the model as the a priori SEM can be seen as a good fit with the experimental data.

## 5. Discussion

The objective of this study was to examine, on the one hand, the changes in periprosthetic BMD of the femur and tibia following TKA. On the basis of the published literature, a decrease in BMD was expected, particularly for the region of the distal femur. On the other hand, the correlation between periprosthetic BMD of the distal femur and the variables iMVF, lean mass, step count, and BMI was analysed. The aim was to identify the relevant predictors for explaining the variance in periprosthetic BMD in order to make appropriate recommendations for preventive measures to combat peri-implant bone loss.

### 5.1. Changes in BMD

We found a significant decrease in periprosthetic BMD of the distal femur 3 months after surgery. This result corresponds with previous findings. There is conclusive evidence in the literature concerning periprosthetic bone loss in the distal femur after TKA [1–8, 11–21]. However, researches published for changes in periprosthetic BMD of the tibia are contradictory [4–6, 17, 22, 23]. The findings of this study did not show any significant changes and concur with the results of, for instance, Soininvaara et al. and Kamath et al. [6, 17]. The present study provides further evidence that there is no change in periprosthetic BMD in the tibia following TKA. The clinical relevance of periprosthetic bone loss remains unexplained, however [1]. The postulated correlation between bone loss caused by stress shielding and periprosthetic fracture is speculative [12, 20, 22, 26] and should be the subject of future research.

### 5.2. Predictors of BMD

The results of the SEM have shown that 55% of the BMD variance is explained by the model. Relevant direct effects on BMD were only evidenced by the variable lean mass. Lean mass accounts for 39% (stepwise regression) and 38% (SEM) of the variance in periprosthetic BMD, respectively. The hypotheses for the direct effects of the variables iMVF, step count, and BMI could not be confirmed.

Various studies demonstrated that muscle mass is related to bone mass [32–38]. In some studies lean mass even accounted for the highest proportion of the variance in BMD [32, 33]. As part of this study, for the first time the relationship between lean mass of the thigh and periprosthetic BMD of the distal femur was examined. Based on findings of previous studies, a positive correlation between the two variables was postulated [32–38]. The results confirm the assumptions and show a significant direct positive effect of lean mass on BMD. Moreover, lean mass was the only significant predictor with the highest variance clarification. A large muscle mass of the thigh thus has a highly protective effect on peri-implant BMD in the region of the distal femur.

The correlation between muscle strength and BMD has not been conclusively proven. Studies have shown a negative relationship between muscle strength and the risk of falling, the impact severity of falls, and the risk of fracture [39, 63, 64]. As, in turn, a lower BMD is associated with an increased risk of fracture [27, 28], a potential correlation between muscle strength and BMD has a high clinical relevance. Existing studies to date focused primarily on the correlation between muscle strength and the BMD of the proximal femur. However, the results are contradictory. On the one hand, significant positive correlations between the variables were found [39, 42, 65, 66] and, on the other hand, no significant results were observed [35]. Segal et al. and Owings et al. also found significant positive correlations between the muscle strength of the lower extremities and BMD in older people [32, 67]. However, when the authors normalised muscle strength to height [32, 67] and to lean mass [32], the correlation was no longer significant. Segal et al. came to the conclusion that the varying results from the studies were due to lean mass not being taken into consideration [32]. The model used for clarifying the variance in periprosthetic BMD in this study took both lean mass and body weight into consideration and did also show no correlation between muscle strength and BMD.

The positive correlation between BMI and periprosthetic BMD could also not be confirmed [41, 42]. The regression analysis and the SEM showed no correlation between the variables. Contrary to assumptions, high body weight does not appear to have a bearing on high periprosthetic BMD. The mechanical stimulus from body weight may thus not be high enough for peri-implant adaptive processes in the bones.

The patients' daily physical activity was quantified by means of the step count and showed no significant correlation with the BMD. This result corresponds with the findings on the correlation between daily physical activity and the BMD of the proximal femur [32, 40, 41]. The most recent Cochrane Review on the effectiveness of different intervention measures on BMD does not show any effect on neither the BMD of the proximal femur nor frequency of fractures if low-intensity training exercises using own body weight (e.g., walking and tai chi) are undertaken [31]. Both the published literature and the result of this study lead to the conclusion that normal day-to-day activity does not constitute a powerful stimulus for adaptive processes in bones. However, the results of a recently published study by Muir et al. counter this theory [43]. According to the study, a significant increase in BMD of the hips can be expected for women over 75 years of age who increase their daily activity levels by more than 2 h per week. These contradictory results may be due to using different methods to assess the levels of physical activity and signal a need for further research.

### 5.3. Limitations

Given the background of methodological difficulties, the findings of our study should be interpreted with caution. The study was limited by the small sample size. The post hoc power coefficients for changes in BMD of the tibia were low (power < 0.42) (Table 4). Thus, an alternative explaining for the nonsignificant findings may be a lack of power due to small sample sizes. Furthermore, SEM generally calls for large sample sizes [59] and the results may also be constrained, due to the low sample size. A reason for this was that the financial support for this project was for a limited period of time, which did not allow the recruitment period to be extended.

Furthermore, indicators of mechanical complications such as inappropriate adjustments of the implant as well as mechanical outcome measures have not been evaluated but may have an influence on BMD. Given this methodological limitation, however, further studies should be carried out to get statistically secure assertions.

## 6. Conclusions

This study analysed, in a first step, the changes in periprosthetic BMD of the femur and tibia following TKA. In a second step, for the first time, the effects of different predictors on periprosthetic BMD of the distal femur were analysed. Variables of particular interest were those whose severity can be modified by different exercise interventions, that is, iMVF, lean mass, step count, and BMI. The aim was to identify the effects of these predictors on BMD in order to make appropriate recommendations for preventive exercise interventions to combat peri-implant bone loss.

The BMD of the distal femur decreased significantly, while no changes were found in BMD of the proximal tibia. Furthermore, the variable lean mass proved to be the only significant predictor of BMD. This leads to the assumptions that a large muscle mass with accompanying distribution of high mechanical load in the bones can contribute to local changes of periprosthetic BMD. Thus, a large muscle mass of the thigh has a high protective effect on peri-implant BMD. As the muscle mass is a predictor which can be modified by intervention measures, some relevant practical implications for preventing peri-implant bone loss may be derived from this finding. Concrete recommendations for use in practice therefore include movement therapy measures which have the aim of maintaining or building up the muscle mass of the lower extremity. The results of this study thus make clear the relevance of movement therapy as part of early postsurgery rehabilitation after a TKA.

## Figures and Tables

**Table 1:** Intrasession reliability for the isometric maximal voluntary force (N) of the leg extensor muscles.

Trial 1 Mean (SD)	Trial 2 Mean (SD)	Mean difference (95% CI)	SD_Diff_	TE (95% CI)	CV% (95% CI)	ICC (95% CI)
1407 (551)	1371 (529)	−36 (−94; 23)	105	75 (55; 118)	6.1 (4.4; 9.7)	0.984 (0.953; 0.995)

SD_Diff_, SD of the difference between sessions 1 and 2; TE, typical error; CV%, coefficient of variation; ICC, intraclass correlation coefficient.

**Table 2:** Demographic and clinical subject characteristics. Values are presented as mean (standard deviation) or numbers (%).

Variable	*n* = 23
Age, yrs	67.7 (8.3)
Sex, men	15.0 (65.2%)
Weight, kg	86.2 (8.2)
Height, m	1.70 (0.10)
BMI, kg/m^2^	29.8 (2.4)
Affected side, right	11.0 (47.8%)
TKA contralateral side	7.0 (30.4%)
THA contralateral side	1.0 (4.3%)
THA ipsilateral side	3.0 (13.0%)
Pretest, postoperative day	9.9 (1.1)
Posttest, postoperative day	93.1 (9.1)

BMI, body mass index; TKA, total knee arthroplasty; THA, total hip arthroplasty.

**Table 3:** Variables used in multivariate analysis. Values are presented as mean (standard deviation) or numbers (%).

Variable	
BMD, g/cm^2^	0.78 (0.34)
iMVF, N	1119.6 (414.4)
Lean mass, kg	5.54 (0.87)
Step count, *n*	37193 (12089)
BMI, kg/m^2^	29.80 (2.40)

BMD, periprosthetic bone mineral density; iMVF, isometric maximum voluntary force; BMI, body mass index.

**Table 4:** Changes of periprosthetic bone mineral density. Values are presented as mean (standard deviation).

Variable	Pretest (*n* = 23)	Posttest (*n* = 23)	Mean difference (95% CI)	*P*	*d*_*z*_	Power	CHBMD (%)
ROI-T1, g/cm^2^	0.93 (0.15)	0.95 (0.22)	0.02 (−0.05; 0.09)	0.629	0.10	0.08	1.70 (16.58)
ROI-T2, g/cm^2^	0.93 (0.14)	0.89 (0.22)	−0.04 (−0.11; 0.03)	0.271	0.21	0.16	−4.04 (17.19)
ROI-T3, g/cm^2^	1.04 (0.12)	0.98 (0.18)	−0.05 (−0.12; 0.02)	0.135	0.38	0.41	−5.30 (16.38)
ROI-F4, g/cm^2^	0.98 (0.11)	0.78 (0.34)	−0.20 (−0.34; −0.06)	0.004^**^	0.67	0.86	−19.65 (32.14)

ROI, region of interest; T, tibia; F, femur; 95% CI, 95% confidence interval; *d*_*z*_, Cohen's *d* effect size; CHBMD, percentage change of periprosthetic bone mineral density.

^**^A significant difference (*P* ≤ 0.010).

**Table 5:** Correlation matrix.

Variable	1	2	3	4	5
(1) BMD	1				
(2) iMVF	0.620^**^	1			
(3) Lean mass	0.628^**^	0.638^**^	1		
(4) Step count	0.326	0.216	0.241	1	
(5) BMI	−0.333	−0.255	−0.117	0.011	1

BMD, periprosthetic bone mineral density; iMVF, isometric maximum voluntary force; BMI, body mass index.

^**^A significant correlation (*P* ≤ 0.010).

**Table 6:** Total standardised and nonstandardised regression coefficients for structural equation modeling.

	Standardised coefficients	Nonstandardised coefficients			*P*
	*β*	*b*	SE	C.R.	
Lean mass*←*step count	0.242	0.000	0.000	1.181	0.238
Lean mass*←*BMI	−0.120	−0.043	0.074	−0.584	0.559
iMVF*←*lean mass	0.598	285.55	78.757	3.626	<0.001^**^
iMVF*←*step count	0.074	0.003	0.006	0.453	0.650
iMVF*←*BMI	−0.186	−32.132	27.645	−1.162	0.245
BMD*←*iMVF	0.286	0.000	0.000	1.480	0.139
BMD*←*lean mass	0.377	0.149	0.074	1.998	0.046^*^
BMD*←*step count	0.176	0.000	0.000	1.179	0.238
BMD*←*BMI	−0.217	−0.031	0.021	−1.456	0.145

BMI, body mass index; iMVF, isometric maximum voluntary force; BMD, bone mineral density; *β*, standardised regression coefficient; *b*, nonstandardised regression coefficient; SE, standard error of the estimate; sr^2^, squared semipartial correlation.

^*^A significant correlation (^*^ *P* ≤ 0.050; ^**^ *P* ≤ 0.010).

**Table 7:** Standardised indirect effects for structural equation modeling.

Variable	Indirect effects			
	BMI	Step count	Lean mass	iMVF
Lean mass	0.000	0.000	0.000	0.000
iMVF	−0.072	0.145	0.000	0.000
BMD	−0.119	0.154	0.171	0.000

BMI, body mass index; iMVF, isometric maximum voluntary force; BMD, periprosthetic bone mineral density.
